# Nanoparticles and cars - analysis of potential sources

**DOI:** 10.1186/1745-6673-7-13

**Published:** 2012-06-22

**Authors:** Stefanie Uibel, Masaya Takemura, Daniel Mueller, David Quarcoo, Doris Klingelhoefer, David A Groneberg

**Affiliations:** 1 Institute of Occupational, Social and Environmental Medicine, Goethe-University, Frankfurt, Germany

## Abstract

Urban health is potentially affected by particle emissions. The potential toxicity of nanoparticles is heavily debated and there is an enormous global increase in research activity in this field. In this respect, it is commonly accepted that nanoparticles may also be generated in processes occurring while driving vehicles. So far, a variety of studies addressed traffic-related particulate matter emissions, but only few studies focused on potential nanoparticles.

Therefore, the present study analyzed the literature with regard to nanoparticles and cars. It can be stated that, to date, only a limited amount of research has been conducted in this area and more studies are needed to 1) address kind and sources of nanoparticles within automobiles and to 2) analyse whether there are health effects caused by these nanoparticles.

## Introduction

**Figure 1:**
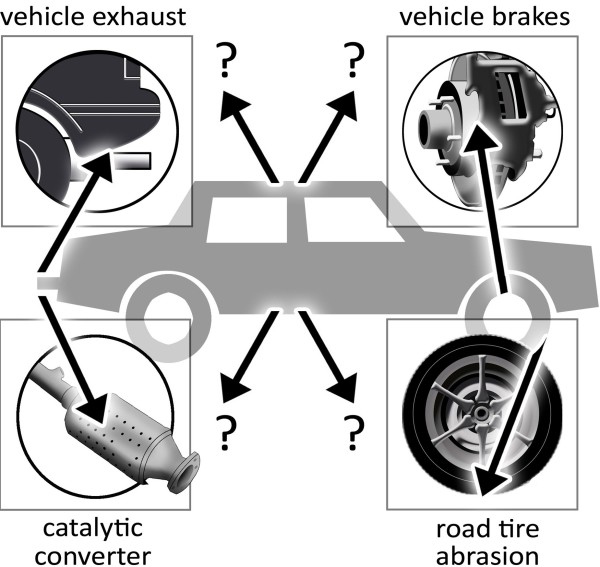
Generation of nanoparticles at different automobile stages.

Both outdoor and indoor air quality are important features that directly influence individual and public health both in occupational and environmental settings. Thus, numerous studies addressed these issues in the past [1-6]. In previous years a large number of studies focused on automobiles as sources for particulate matter. A few review articles address this issue [7-10]. In contrast, nanoparticle (NP) research has only touched the field of automobile industry at the periphery. So far, it is commonly accepted that NPs may be generated at the vehicle brake, the vehicle exhaust and at road-tire abrasion processes (Figure 1).

Since detailed reviews have not been performed so far, this article intends to summarize recently published data on nanoparticle research in relation to automobiles. Hence, this article deals with the kind of nanoparticles which may arise from traffic as well as their potential sources.

## Palladium nanoparticles: Solution-engineered NPs as a model for health effect studies of automotive particulate pollution

A recent study by Wilkinson *et al.* addressed the use of solution-engineered palladium (Pd) nanoparticles as a model for health effect studies of automotive particulate pollution. The authors state that over 60% of platinum group metals (PGMs) (Ru, Os, Rh, Ir, Pd, and Pt) are used for the production of automobile catalytic converters [11,12]. These converters are constructed by deposition of PGMs on a honeycomb-cordierite substrate covered by a washcoat of cordierite and γ-Al_2_O_3_[11]. There may be health effects due to particles which are emitted from converters, but it is extremely difficult to generate PGM-NPs. Therefore, the authors sought an alternative for the production of these NPs by the use of solution synthesis [11] with the production of dispersions of hydrophilic spherical Pd nanoparticles (Pd-NPs) of uniform shape and size (10.4 ± 2.7 nm) in one step by Bradley's reaction (solvothermal decomposition in an alcohol or ketone solvent). A similar approach also provided mixtures of Pd-NPs and nanoparticles of non-redox-active metal oxides, such as Al_2_O_3_. The authors furthermore studied particle aggregation in applied media by DLS and NP tracking analysis.

Three years earlier, a group from Munich reported the preparation and characterization of Pd/Al_2_O_3_ and Pd nanoparticles as standardized test material for chemical and biochemical studies of traffic related emissions [13]. Specifically, two series of Pd particles were prepared: Pd NPs with 2–4 nm dispersed on aluminium oxide particles of a diameter range between 0.1 to 30 μm and "Pd-only" NPs of 5–10 nm in diameter [13]. The Pd/alpha-Al_2_O_3_ particles were reported to be very similar to particles emitted from catalytic converters by mechanical abrasion. The Pd-only particles were suggested to be useful *e.g.* for exposure studies in which the presence of aluminium could lead to interferences when studying biological and biochemical effects [13]. In contrast to the study by Leopold *et al.* who characterized the NPs using transmission electron microscopy (TEM), high resolution transmission electron microscopy (HRTEM), selective area diffraction (SAD), laser granulometry and graphite furnace atomic absorption spectrometry (GFAAS) for the measurement of Pd concentrations [13], the group of Wilkinson *et al.* also performed toxicological experiments in order to assess putative health effects of the produced Pd NPs and nanocomposite mixtures. For this purpose, they used human primary bronchial epithelial cells (PBEC) and human alveolar carcinoma cell line (A549) as model system for NP-exposure [11]. They reported that a cellular uptake of Pd nanoparticles was only visible in PBEC, as determined by TEM. However, they found pronounced and dose-dependent effects on cellular secretion of soluble biomarkers both in PBECs and A549 cells and a decreased responsiveness of human epithelial cells to the pro-inflammatory cytokine TNF-α [11]. Interestingly, when cells were incubated with higher doses of the Pd nanoparticles, induction of apoptosis and caspase activation were observed present in PBEC but not in A549 cells [11]. In summary, the authors concluded that this mode of Pd NP generation is applicable to study the effects of Pd NPs. It is important to realize that this study cannot be used for an exact toxicological assessment for traffic-related health effects of Pd NPs since it is too preliminary.

## Gold nanoparticles: Subchronic inhalation toxicity studies

Besides the use of gold nanoparticles (NPs) in cosmetics, food packaging, beverages, toothpaste, and lubricants, they may also be present in automobiles [14]. Since only a few studies have reported data on the toxicology of gold NPs so far, Sung *et al.* recently performed subchronic inhalation studies of gold nanoparticles in Sprague Dawley rats [14]. Next to a control group, a low-dose (2.36 × 104 particle/cm^3^, 0.04 μg/m^3^), a middle-dose (2.36 × 105 particle/cm^3^, 0.38 μg/m^3^), and a high-dose (1.85 × 106 particle/cm^3^, 20.02 μg/m^3^) group of animals were exposed for 6 hours/day, 5 days/week, for 90-days in a whole-body inhalation chamber. The average diameter of the gold NPs was 4–5 nm. The authors studied mortality and observed clinical symptoms, body weight, food consumption, and lung function on a weekly basis. Also, necropsy and blood and bronchoalveolar lavage sample analysis were performed at the end of the exposure protocol [14].

The authors reported that within the lung function tests, tidal volume and minute volume showed a tendency – but no significancy – to decrease comparing control and dose groups during the 90-days of exposure [14]. Also, no statistically significant differences were present in cellular differential counts. The microscopic analysis showed an inflammatory infiltrate with a mixed cell type, and increased macrophages in the high-dose group of exposed animals [14]. The tissue distribution analysis demonstrated a dose-dependent accumulation of gold in the lungs and the kidneys [14]. Interestingly, there was a gender-related difference in gold NP content in the kidneys present. The authors conclude that the lungs were the only organ in which dose-related changes in both male and female rats were observed [14].

## Particles released from low-metallic automotive brakes

In April 2011, Kukutschová *et al.* reported a study that focussed on wear particles released from commercially available “low-metallic” automotive brake pads which were subjected to brake dynamometer tests in the experiments [15]. They determined particle size distribution *in situ* and collected the generated particles for further studies. The collected fractions and the original bulk material were analyzed using a multitude of techniques and it was found that airborne wear particles with sizes between 10 nm and 20 μm were released into the air during the experiments. Interestingly, the numbers of nanoparticles (< 100 nm) were by three orders of magnitude larger in comparison to the number of microparticles [15].

Concerning the temperature, the authors stated that size distribution of airborne wear particles generated during brake dynamometer simulations representing sub-urban driving segments varies in dependence on temperature of the friction surface [15]. They found that the generation of wear particles smaller than 500 nm was negligible for cold surfaces, while the concentration of nano-sized particles (< 100 nm) gradually increased when the bulk temperature of the rotor approached approximately 300 °C. They also reported that thermal analysis of milled bulk brakes performed in oxidative and inert atmospheres showed a heterogeneous ignition mechanism of carbonaceous particles [15]. The ignition temperature is approximately 300 °C, which correlates with the rotor temperature when the maximum number of finest particles was generated [15].

In total, the analysed friction composite was a multicomponent heterogeneous material. However, the dominant elements in the finest wear particle fractions were Fe and C. It was not easy to distinguish Cu, Sn and other metals/oxides in diffraction measurement. Further PIXE analysis revealed the presence of Fe, Cu, Sn, Zn and S in all fractions [15].

Wear particle analysis was performed using Raman microspectroscopy and showed the presence of carbon black and graphitic particles. Also, TEM-diffraction analysis identified the presence of maghemite (γ-Fe_2_O_3_), magnetite (FeO·Fe_2_O_3_) and hematite (α-Fe_2_O_3_) in collected nano fractions. All wear particle fractions contained nano-sized particles down to 20 nm in diameter in the form of agglomerates [15].

**Figure 2:**
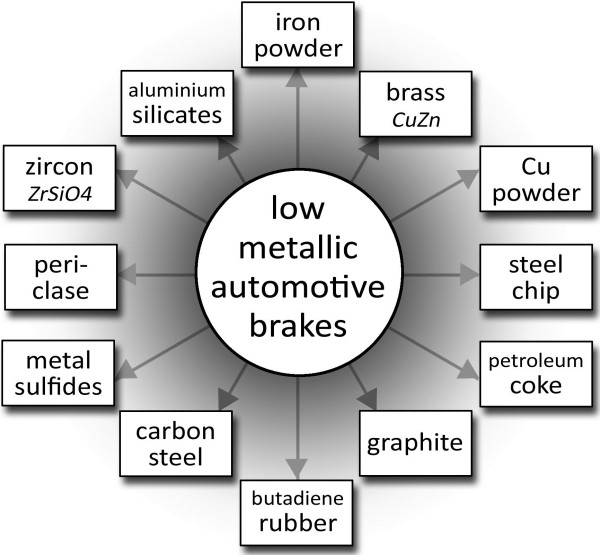
**Easily detectable constituents in the bulk brake lining sample as reported by Kukutschová *et al .***[15.]

The authors concluded that wear of low-metallic friction composite produces airborne NPs. These NPs contain carbon black and a variety of metallic compounds (Figure 2). It needs to be stated that a comprehensive evaluation of particles which contribute to air pollution as presently performed requires a combination of several microscopic techniques and chemical analyses to determine the real size of individual particles present in particulate matter [15].

## Organometallic fuel additives and nanoparticle emissions from gasoline passenger cars

Gidney and colleagues stated in their recent article that the European particle measurement program (PMP) and the new EURO 6 regulations on particle emissions from diesel cars were intended to promote the use of diesel particulate filters (DPFs) but excluded particles smaller than 23 nm because of measurement difficulties in that size range [16]. However, these regulations do not imply that particles below 23 nm are benign and to clarify the fate of organometallic gasoline fuel additives from a gasoline engines, the authors studied particulate number and size distributions in the exhaust line of a typical European family passenger car with a 1.6 L four cylinder spark ignition engine (multi point injection, 4 cylinder, 1.6 L displacement, catalyst volume 1.66 L, European Stage 3 emissions standards) fuelled with standard gasoline and gasoline containing an organometallic additives [16]. Three additives were tested: CH_3_C_5_H_4_Mn(CO)_3_, Fe(Cp)_2_ and PbEt_4_. Two concentrations of CH_3_C_5_H_4_Mn(CO)_3_ were used, 8.3 mg/L (Mn-8) and 18 mg/L (Mn-18) of manganese. These concentrations represent levels that were historically permitted in the U.S. and Canada, respectively. One concentration was used for the FeCp_2_ and PbEt_4_ tests (Fe-8 − 8.4 mg/L and Pb-30 − 31.3 mg/L). This was the same concentration of metal in the fuel as the Mn-8 case [16]. They measured particle size on the exhaust of a car operating on a chassis dynamometer fuelled with standard gasoline and gasoline containing low levels of Pb, Fe, and Mn organometallic additives. Interestingly, when additives were present in the experimental setup, there was a distinct nucleation mode consisting primarily of sub-10 nm nanoparticles [16]. It was found that at equal molar dosing Mn and Fe gave similar NP concentrations at the tailpipe, whereas Pb gave a considerably lower concentration. In further experiments the authors used a catalytic stripper in order to remove the organic component of these particles. It was shown that they were mainly solid and, because of their association with inorganic additives, presumably inorganic [16].

The authors stated that solid nucleation mode NPs of similar size and concentration to those observed in the present study from a gasoline engine with Mn and Fe additives have also been observed from modern heavy-duty diesel engines without after-treatment at idle, but these solid particles are a small fraction of the primarily volatile nucleation mode particles emitted [16]. The authors suggested that solid nucleation mode particles emitted by the diesel engines are likely derived from metal compounds in the lubrication oil, although carbonaceous particles cannot be ruled out. They also stated that most of these solid nanoparticles emitted by both engine types fall below the 23 nm cutoff of the PMP number regulation.

## Conclusion

In view of the current research on nanoparticles, it needs to be stated that the amount of published articles on nanoparticles, which may arise from traffic, is still largely limited. Therefore, modern scientometric tools which are in use for the analysis of other research fields are not applicable in this area [17-29].

As a conclusion, there is a lack of information present regarding a) levels of nanoparticles that are emitted from automobiles b) effects of nanoparticles with regard to the emission levels. There are numerous approaches present which may bring light into this field of environmental sciences. Specifically, sources and levels of different NPs need to be identified and analyzed. Additionally, further toxicological research should be performed about molecular mechanisms, *i.e.* with the use of advanced techniques of biochemistry [30-33] and molecular biology [34-38].

## Competing interests

The authors declare that they have no competing interests.

## Authors' contributions

SU, MT, DM, DQ, DK, DAG have made substantial contributions to the conception and design of the review, acquisition of the review data and have been involved in drafting and revising the manuscript. All authors have read and approved the final manuscript.
